# Synthesis of Mg-Fe-Cl hydrotalcite-like nanoplatelets as an oral phosphate binder: evaluations of phosphorus intercalation activity and cellular cytotoxicity

**DOI:** 10.1038/srep32458

**Published:** 2016-09-01

**Authors:** Yung-Feng Lung, Ying-Sui Sun, Chun-Kai Lin, Jun-Yen Uan, Her-Hsiung Huang

**Affiliations:** 1 Department of Materials Science and Engineering, National Chung Hsing University, 145 Xingda Rd., South Dist., Taichung 402, Taiwan, ROC; 2 Department of Dentistry, National Yang-Ming University, 155, Sec.2, Linong Street, Taipei 112, Taiwan, ROC; 3 Green Energy and Environment Research Laboratories, Industrial Technology Research Institute, 195 Sec. 4, Chung-Hsing Road, Hsin-Chu 31040, Taiwan, ROC; 4 Department of Bioinformatics and Medical Engineering, Asia University, 500 Lioufeng Rd., Wufeng, Taichung 413, Taiwan 500, ROC; 5 Department of Medical Research, China Medical University Hospital, 2 Yude Rd., Taichung 407, Taiwan, ROC

## Abstract

The patients with end-stage of renal disease (ESRD) need to take oral phosphate binder. Traditional phosphate binders may leave the disadvantage of aluminum intoxication or cardiac calcification. Herein, Mg-Fe-**Cl** hydrotalcite-like nanoplatelet (HTln) is for the first time characterized as potential oral phosphate binder, with respect to its phosphorus uptake capacity in cow milk and cellular cytotoxicity. A novel method was developed for synthesizing the Mg-Fe-**Cl** HTln powder in different Mg^2+^: Fe^3+^ ratios where the optimization was 2.8:1. Addition of 0.5 g Mg-Fe-**Cl** HTln in cow milk could reduce its phosphorus content by 40% in 30 min and by 65% in 90 min. In low pH environment, the Mg-Fe-**Cl** HTln could exhibit relatively high performance for uptaking phosphorus. During a 90 min reaction of the HTln in milk, no phosphorus restoration occurred. *In-vitro* cytotoxicity assay of Mg-Fe-**Cl** HTln revealed no potential cellular cytotoxicity. The cells that were cultured in the HTln extract-containing media were even more viable than cells that were cultured in extract-free media (blank control). The Mg-Fe-**Cl** HTln extract led to hundred ppm of Mg ion and some ppm of Fe ion in the media, should be a positive effect on the good cell viability.

Hydrotalcite-like nanoplatelets (HTln) belong to a class of anionic clay minerals that are also known as layered double hydroxides. HTln has the general formula 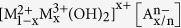
·mH_2_O^1^, where M^2+^ and M^3+^ are divalent and trivalent metal cations, respectively, in the octahedral positions within the hydroxide layers. A^n−^ is an anion (such as CO_3_^2−^, SO_4_^2−^, Cl^−^) and x can take values between 0.2 and 0.33^1^. According to Cavani^1^, the HTln structure contains brucite (Mg(OH)_2_)-like layers in which some of the divalent cations have been replaced by trivalent ions, forming positively charged sheets. This charge is compensated by the intercalation of anions in the hydrated interlayer regions^1^,^2^.

HTln exhibit anion-exchangeability, referring to the uptake of new anions from its environment^3^. On account of this unique characteristic of HTln, several investigations have reported that HTln can probably be used as an oral phosphate binder^4^,^5^,^6^. Phosphorus control is a primary goal in the care of patients with end-stage renal disease (ESRD)^7^. Dietary phosphorus restriction and hemodialysis often fail adequately to control serum phosphorus. Western diet includes 1000 to 1200 mg of dietary phosphate, of which approximately 800 mg is ultimately absorbed daily^8^. The phosphorus in food additives (such as monocalcium phosphate or sodium phosphate) potentially accounts for an additional 500 mg per day^9^. Therefore, phosphate binders are routinely prescribed for patients to reduce their intestinal absorption of phosphate^10^. Among currently used oral phosphate binders, aluminum-containing binders were extensively used until the mid-1980s^11^, when they were largely abandoned because of potential aluminum intoxication^10^,^12^. Calcium-based phosphate binders (calcium carbonate and calcium acetate) are therefore commonly prescribed in the United States^11^. Nevertheless, the intake of large doses of calcium may contribute an excess calcium load and cause cardiac calcification^13^. With respect to the above, aluminum-free and calcium-free phosphate binders are critical for reducing the absorption of dietary phosphorus. The ideal phosphate binder must efficiently bind phosphate, undergo minimal systemic absorption, have few side effects, have a low pill burden, and be inexpensive^11^. Several new products such as sevelamer hydrochloride (Renagel)^14^, fermagate (Mg-Fe-CO_3_ HTln)^15^ and fosrenol (lanthanum carbonate)^11^,^16^ have been reportedly utilized in short-term clinical research. Sevelamer hydrochloride (Renagel) is a synthetic aluminum-free and calcium-free phosphate binder^17^ that is efficient and therefore used^18^ in the management of hyperphosphatemia in ESRD^19^. However, the cost of taking sevelamer hydrochloride (Renagel) in phosphate binder therapy is approximately six times that of taking the traditional binder, calcium acetate (PhosLo), imposing a cost on patients and the nation^10^. Moreover, 38% of patients who take Renagel suffer from the side effects of dyspepsia and diarrhea or constipation^20^. Roberts *et al*.^21^ compared the modern phosphate binders sevelamer hydrochloride (Renagel), Mg-Fe-CO_3_ HTln and La_2_(CO_3_)_3_ with traditional binders Al(OH)_3_, Mg(OH)_2_ at pH 3 and pH 7, mimicking the conditions of the gastrointestinal tract, in terms of phosphate binding capacity, and found that sevelamer hydrochloride (Renagel) bound 90% of phosphate at pH 3 but only 57% of phosphate at pH 7. Similarly, the phosphate binding performance of La_2_(CO_3_)_3_ is reportedly related to pH^16^, the optimal range of which is pH 3 ~ pH 5^16^,^21^. At pH 7, it bound less than 5% of phosphate^21^, indicating that the poor binding by La_2_(CO_3_)_3_ might have arisen from the narrow range of pH availability^11^,^16^. For comparison, Mg-Fe-CO_3_ HTln reportedly bound 70% of phosphate at pH 3 and only 45% at pH 7. Zhu *et al*.^5^ found that at least ten times as much La_2_(CO_3_)_3_ by weight is required to bind to a degree comparable with that of Mg-Fe-CO_3_ HTln.

Various methods for synthesizing HTln powder have been developed^2^,^6^,^22^,^23^,^24^,^25^,^26^,^27^. Following synthesis, a hydrothermal treatment must be performed. The treatment takes a few hours to several days to yield crystalline Mg-Fe-CO_3_ HTln. The feasibility of rapidly synthesizing crystalline HTln using a simple and efficient method has attracted research attention^28^,^29^,^30^,^31^. Additionally, according to Miyata *et al*.^32^, the order of the affinities of HTln for intercalating anions can be written as chloride ions <phosphate ions <carbonate ions. Therefore, Mg-Fe HTln with Cl^−^ intercalation have higher phosphate ion exchangeability than those with CO_3_^2−^ intercalation, and so may exhibit greater phosphate binding capacity with a smaller pill burden than currently available Mg-Fe-CO_3_ HTln. The following researchers studied the production of Mg-Fe-Cl HTln but did not test them as oral phosphate binders. Tong *et al*.^33^, Meng *et al*.^2^ and Caporalea *et al*.^34^ mixed MgCl_2_ and FeCl_3_ in a concentrated alkaline aqueous solution under N_2_ to form a reddish-brown suspension, which had to be hydrothermally treated at room temperature for 24 h^34^ or 100 °C for 6 h^2^ to yield crystalline Mg-Fe-Cl HTln. Chitrakar *et al*.^35^ prepared Mg-Fe-Cl HTln powder by mixing solid MgO with FeCl_3_ solution at 30 °C. This method took more than two days to yield crystalline Mg-Fe-Cl HTln^35^. The HTln took up bromide ions from solutions of pollutants^35^.

Research progresses have been made in the production and application of Mg-Fe-Cl HTln. However, the above methods take a long time to implement. Moreover, the phosphate binding performance of Mg-Fe-Cl HTln, as an oral phosphate binder, in cow milk and in concentrated phosphate solution has not yet been evaluated. This work develops efficient process for synthesizing crystalline Mg-Fe-Cl HTln in 2 hr at 50 °C. The phosphate binding performance of the Mg-Fe-Cl HTln was evaluated by performing phosphate uptake tests in aqueous KH_2_PO_4_ solution (pH 3 and pH 6) and cow milk at various pH values. For comparison, the binding performance of a commercial phosphate binder (sevelamer hydrochloride (Renagel)) in cow milk was also evaluated. An *in vitro* cellular cytotoxicity assay of the Mg-Fe-Cl HTln was also performed.

## Results and Discussion

### Characterization of synthesized Mg-Fe-Cl HTln

**Figure 1:**
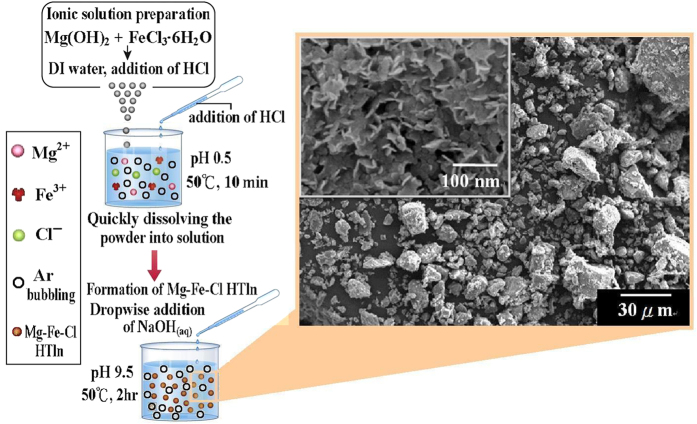
Schematic depiction for the preparation of Mg-Fe-Cl hydrotalcite-like nanoplatelet (HTln).

**Figure 2:**
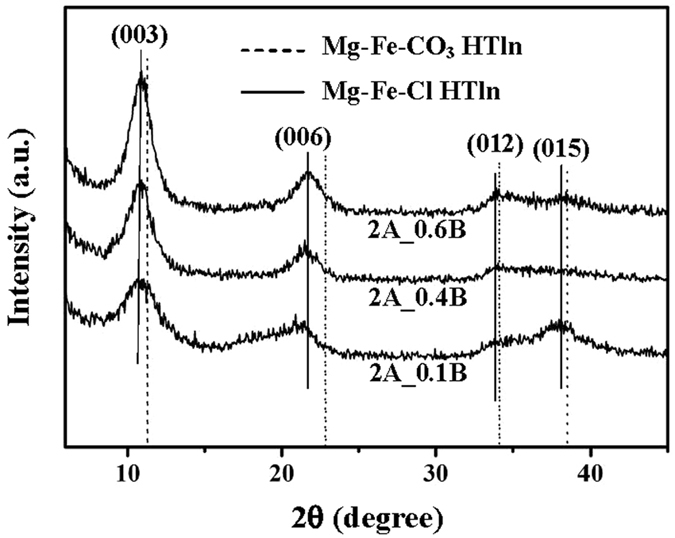
X-ray powder diffraction pattern of Mg–Fe–Cl HTln 2A_0.1B, 2A_0.4B and 2A_0.6B.

Figure 1 show schematic diagram of formation of Mg-Fe-Cl hydrotalcite-like nanoplatelet (HTln). The inset in the SEM image shows the nanoplatelet structure of HTln. The preparation conditions for the synthesis of Mg-Fe-Cl HTln have been illustrated in Table 1. Herein, the SEM image in Fig. 1 is the synthesized Mg–Fe–Cl HTln that was made from the condition of 2A_0.6B. Figure 2 presents the X-ray powder diffraction patterns of the synthesized Mg-Fe-Cl HTln powder. The reflection peaks at 10.90° and 21.65° were identified as basal reflections (003) and (006) from hydrotalcite. The (003) basal spacing, 8.11 Å, of the Mg-Fe-Cl HTln is similar to that of the Mg-Fe-Cl HTln obtained by conventional methods^2^,^36^. The (003) basal spacing (8.11 Å) of the HTln herein exceeds that of Mg-Fe-CO_3_ HTln (7.97 Å)^2^,^36^. As presented in Fig. 2, each of the X-ray intensity peaks of the Mg-Fe-Cl HTln were shifted toward lower angles from corresponding X-ray peaks of Mg-Fe-CO_3_ HTln, revealing that the carbonate ion was not the main intercalation anion. Although preventing contamination from carbonate ions in the preparation of HTln is difficult^1^, the synthetic method that was developed herein successfully minimizes carbonate contamination. Based on the X-ray reflection patterns in Fig. 2, the HTln that was made from 2A_0.6B exhibited much greater crystallinity than those made from 2A_0.1B and 2A_0.4B. Table 2 presents the Mg^2+^, Fe^3+^, Cl^−^ and Na^+^ contents (in at.%), obtained by XPS, of the samples 2A_0.1B, 2A_0.4B and 2A_0.6B. The chemical contents of each sample were calculated from its corresponding Mg 2p, Fe 2p3, Cl 2p and Na 1s XPS spectra. In Table 2, 2A_0.1B contained 0.8% Cl^−^; 2A_0.4B contained 1.2% Cl^−^, and 2A_0.6B contained 2.3% of Cl^−^. Importantly, sodium was not detected in the various samples, supporting the finding that Cl^−^ was not associated with the residual NaCl but was intercalated in the HTln. The general formula for HTln is of the form [M_1−**x**_^2+^M_*x*_^3+^(OH)_2_]^*x*+^(A_*x/n*_^*n−*^)·*n*H_2_O^1^, where x equals to M^2+^/(M^2+^+M^3+^). Cavani *et al*.^1^ stated that, for a natural HTln, x is typically 0.25. Table 2 presents the x values of 2A_0.1B, 2A_0.4B and 2 A_0.6B. The x value of 2A_0.6B is 0.26. Goh *et al*.^37^ found that the best crystalline HTln phase was generally obtained with an M^2+^/M^3+^ ratio of 3:1, which maximized the sorption of oxyanions. In this study, this ratio for 2A_0.6B is 2.8:1 which is close to the optimal ratio. Therefore, based on the synthesis method explored herein, 2A_0.6B was used to synthesize Mg-Fe-Cl HTln for the following phosphate binding experiments.

### Uptake of PO_4_^3**−**^ from aqueous KH_2_PO_4_

**Figure 3:**
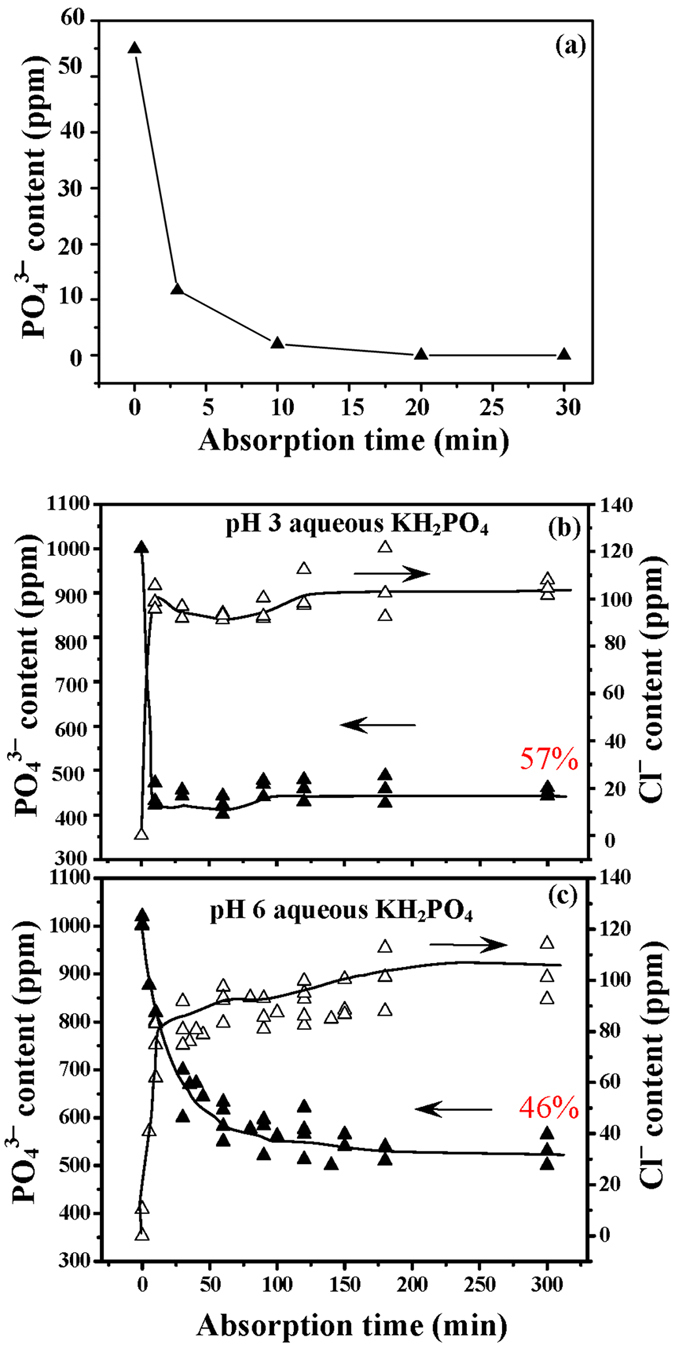
(**a**) PO_4_^3−^ sorption by 0.2 g Mg–Fe–Cl HTln in 100 ml aqueous KH_2_PO_4_ (original 55 ppm of PO_4_^3−^); (**b**,**c**) PO_4_^3−^ sorption and Cl^−^ desorption when immersing 0.2 g Mg–Fe–Cl HTln in 100 ml aqueous KH_2_PO_4_ (original 1000 ppm of PO_4_^3−^), where (**b**) sorption and desorption experiments being conducted at pH 3, (**c**) sorption and desorption experiments being conducted at pH 6. The pH values of the solutions were maintained by dropwises of HNO_3_(aq).

When 0.2 g HTln powder is added to 100 ml aqueous KH_2_PO_4_ (which initially contained 55 ppm of PO_4_^3−^) at room temperature, as presented in Fig. 3(a), the HTln took up almost all PO_4_^3−^ in the solution in 20 min. To determine the PO_4_^3−^-uptaking capacity of the Mg-Fe-**Cl** HTln, 0.2 g of the HTln powder was added to 100 ml aqueous KH_2_PO_4_ with a PO_4_^3−^ initial content of 1000 ppm (Fig. 3). Figure 3(b) reveals that when 0.2 g of Mg-Fe-**Cl** HTln was added to the 100 ml aqueous KH_2_PO_4_ maintained at pH 3, with an original PO_4_^3−^ concentration 1000 ppm, the solution only ~430 ppm of residual PO_4_^3−^ (57% phosphate uptake) after 10 min, and almost no more PO_4_^3−^ was taken up from 10 min to 300 min. During the same experimental period, the chloride anion was de-intercalated from the Mg–Fe–**Cl** HTln, providing evidence of anion intercalation and de-intercalation. Figure 3(c) shows that 0.2 g Mg-Fe-Cl HTln in 100 ml aqueous KH_2_PO_4_ with 1000 ppm of PO_4_^3−^ at pH 6 took up PO_4_^3−^, reducing the phosphate content to ~540 ppm in 100 min, and almost no more PO_4_^3−^ was taken up from 100 min to 300 min. The phosphate uptake capacity of the Mg-Fe-**Cl** HTln herein in aqueous KH_2_PO_4_ at pH 6 was ~46%, lower than that of the HTln at pH 3. Zhu *et al*.^5^ investigated phosphate binding at pH 3 by adding 0.025 g **CO**_**3**_^**2−**^−intercalated Mg-Fe HTln (i.e., Mg-Fe-**CO**_**3**_ HTln) to 5 ml phosphate solution (equivalent to adding 0.5 g Mg-Fe-**CO**_**3**_ HTln to 100 ml phosphate solution). The Mg-Fe-**CO**_**3**_ HTln powder in phosphate solution reduced the phosphate content of the solution by only 24%^5^. The proportional phosphate content reduction was even lower at 13% when the solution pH was maintained at pH 7^5^. Evidently, the phosphate binding capacity of the Mg-Fe-**Cl** HTln in KH_2_PO_4_ solution of this study was much higher than that of the Mg-Fe-**CO**_**3**_ hydrotalcite compound by Zhu *et al*.^5^. According to Kazama^38^, hydrotalcite-like compounds are typically unstable under acidic conditions, and this instability may be problematic when they are used as oral phosphate binders. Hence, Seida and Nakano^39^ examined the effect of initial pH of aqueous PO_4_^3−^ on both the concentration of dissolved metal cations and the removal of phosphate by Mg-Fe-**CO**_**3**_ HTln. Carbonate ions that are intercalated in hydrotalcite compound are difficult to exchange with the other anions in the environment owing to their high affinity to the hydrotalcite^40^. Therefore, Iyi *et al*.^40^ and Lung *et al*.^28^ synthesized Mg-Al-**Cl** HTln powder^40^ and Mg-Al-**Cl** HTln thin film^28^ to take up hazardous anions from wastewater. Seida and Nakano^39^ found that the amount of phosphate that was removed by Mg-Fe–**CO**_**3**_ HTln increased as the initial pH of the aqueous PO_4_^3−^ decreased below pH 3. Dissolution of the Mg-Fe-**CO**_**3**_ HTln releases cations and/or hydroxides, yielding a final pH of the PO_4_^3−^−containing solution of as high as pH 9^39^. Therefore, instead of anion exchange between the intercalated CO_3_^2−^ and the PO_4_^3−^, the released cations and/or hydroxides which increase the solution pH act as coagulants and/or precipitants in phosphate removal^39^. Herein, Mg-Fe-**Cl** HTln was studied. As shown in Fig. 3(b), Mg-Fe-**Cl** HTln powder in aqueous KH_2_PO_4_ at pH 3 exhibited an excellent ability to take up phosphate from acidic solution, reducing the phosphate concentration from ~1000 ppm down to ~450 ppm. Importantly, during the 5 hr uptake of phosphate in aqueous KH_2_PO_4_ with pH fixed at pH 3, the HTln was stably maintained the phosphate content of the aqueous KH_2_PO_4_ at ~450 ppm throughout the experimental period, suggesting that the HTln structure did not disintegrate under acidic conditions, so phosphate restoration did not occur. The concentration of chloride increased during this period, indicating direct evidence of anion exchange.

### Swelling test and uptake of dietary phosphorus in milk: Mg-Fe-Cl HTln *vs*. Renagel

**Figure 4:**
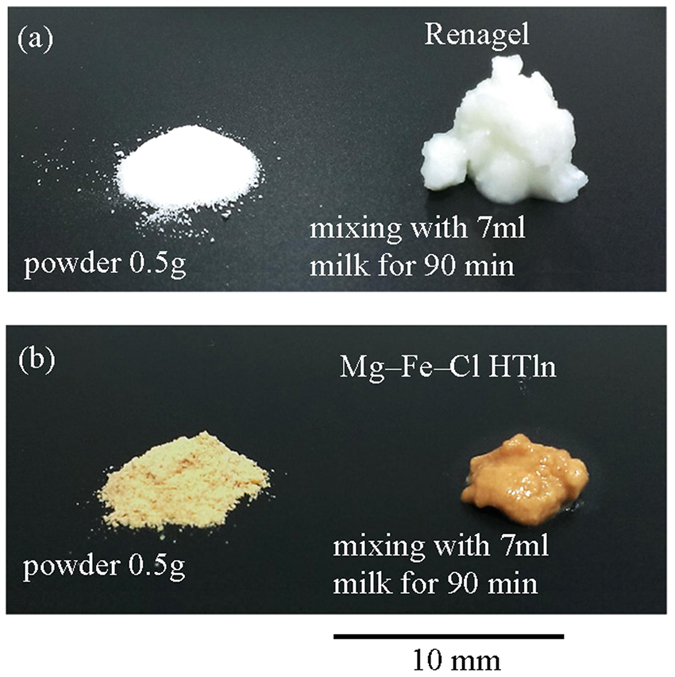
(**a**) 0.5 g Renagel powder mixing with 7 ml milk, showing a relatively large volume expansion; (**b**) 0.5 g Mg–Fe–Cl HTln powder mixing with 7 ml milk, indicating almost no expansion.

In at least three *in vitro* swelling tests, 0.5 g Renagel powder has been shown to swell by approximately 1150% in volume when placed in milk. A similar swelling phenomenon has reported elsewhere^14^. In an identical test, 0.5 g of Mg-Fe-**Cl** HTln powder was found to swell by only ~33 vol.% when placed in milk. Figure 4 displays images of Renagel (Fig. 4(a)) and Mg-Fe-**Cl** HTln (Fig. 4(b)) before and after mixing with 7 ml of milk for 90 min. As shown, the slurry of 0.5 g Renagel had a greater volume than that of 0.5 g Mg-Fe-**Cl** HTln.

**Figure 5:**
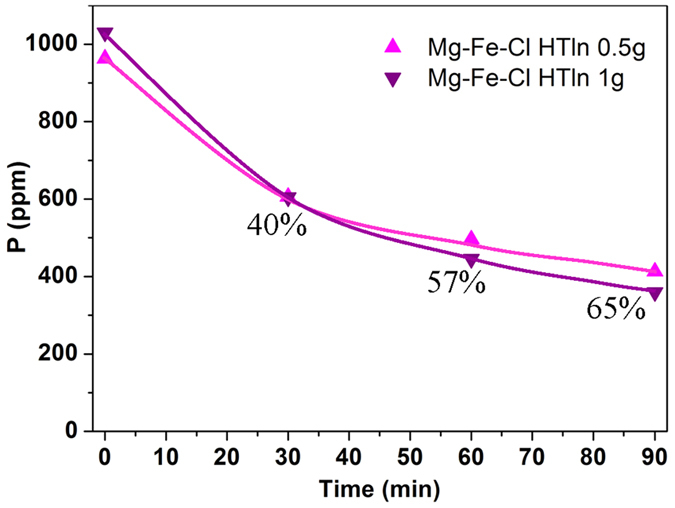
Phosphorus uptake by Mg–Fe–Cl HTln in milk, with the milk’s pH maintained at pH 6 during the experiment by dropwise of HNO_3(aq)_. The percentage in the figure was the phosphorus absorption ratio at 90 min.

Figure 5 presents the dietary phosphorus-binding performance of Mg-Fe-**Cl** HTln in milk at pH 6, which falls in the gastric pH range of 5 to 6.7 (which is the highest recorded pH within 5 min of eating) during a meal^41^. In digestion, the stomach forms chime, which is transferred to the duodenum^42^. The small intestine is critical in phosphate absorption^43^. Therefore, removing most of the phosphate using a phosphate binder during digestion in the stomach is important for ESRD patients. Hunt *et al*.^42^ described how most of a meal was digested in the stomach, which was usually emptied of food in approximately 90 min. Herein, the following experiments were performed for 90 min. A 0.5 g mass of Mg-Fe-**Cl** HTln (added to 25 ml milk at pH 6) efficiently reduced the phosphorus content by approximately 40% in 30 min, and reduced it by ~65% after 90 min, as presented in Fig. 5. Moreover, phosphorus binding by 1 g Mg-Fe-**Cl** HTln (in 25 ml milk at pH 6) is similar in extent to that by the 0.5 g Mg-Fe-**Cl** HTln. According to Roberts *et al*.^21^, the phosphorus binding performance of Renagel (a popular commercial phosphate binder) in pH 7 environments is much lower than that of the Renagel in pH 3. Mg-Fe-**Cl** HTln has similar result, as shown in Fig. 3(b,c), that the HTln in pH 6 solution exhibited a relatively low phosphorous binding performance. Therefore, as the data plots in Fig. 5, samples 0.5 g and 1 g exhibited similar performance to take up phosphorus, which may be due to the negative effect of pH 6 in tested milk. In other words, a larger dose of Mg-Fe-**Cl** HTln cannot exhibit much better phosphorous binding performance than the fewer doses did when the milk was at pH 6 or higher. However, the mechanism that underlines the effect of high pH on decreasing the phosphorous binding performance of Mg-Fe-**Cl** HTln requires further studies. Nevertheless, in real case, ~pH 6 is the highest pH in the beginning of eating during a meal^41^, since gastric acid would subsequently reduce the pH of the food in stomach.

**Figure 6:**
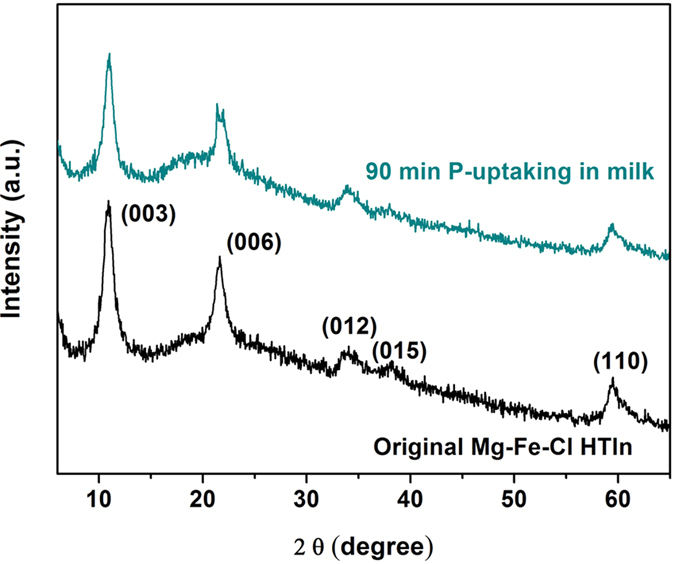
X-ray powder diffraction patterns of the Mg–Fe–Cl HTln before and after phosphate uptake in milk, suggesting that the crystal structure of the HTln was not changed in milk for the 90-min experiment.

**Figure 7:**
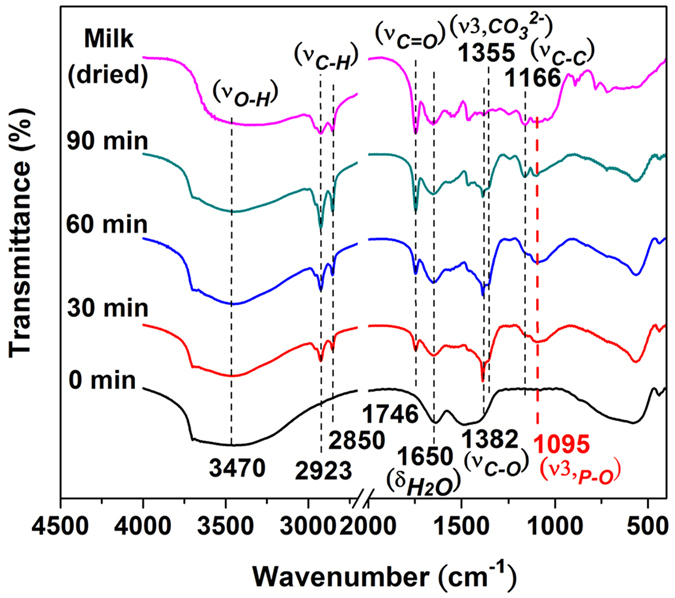
FT-IR spectra of 1 g Mg–Fe–Cl HTln (denoted as 0 min) and the HTln spectra after phosphate uptake in 25 ml milk at pH 6 (denoted as 30 min, 60 min and 90 min). For comparison, the spectrum of dried milk was also shown in this figure. The signal at wavenumber 1095 cm^−1^ was absorption band by P-O bond.

Figure 6 presents the X-ray diffraction patterns of the Mg-Fe-**Cl** HTln before and after the phosphorus binding experiments in milk for 90 min. A 1 g mass of Mg-Fe-**Cl** HTln has the capacity to take up phosphorus in milk, reducing the phosphorus content in milk from 1030 ppm to 359 ppm in 90 min (Fig. 5). As shown in Fig. 6, the X-ray diffraction pattern of the Mg-Fe-**Cl** HTln after the 90 min experiment was similar to that of the original Mg-Fe-**Cl** HTln, suggesting that the uptake of phosphorus from milk did not change the layered structure of the HTln. The chemical bonds in the Mg-Fe-**Cl** HTln after the experiment in milk in different states were identified using FT-IR (as the spectra shown in Fig. 7). The FT-IR spectrum of dried milk powder is also shown for comparison. The band that is centered at 570 cm^−1^ is attributed to *M*–O–*M* vibration^36^,^44^,^45^, which like the *M*–O–H bending at around 446 cm^−1^ 46, involves translational motion of the oxygen cation in the brucite-like layers^47^,^48^. The broad strong absorption band at 3470 cm^−1^ was attributed to the H-bond stretching vibrations of the OH group (ν_O–H_) in the brucite-like layers^22^,^47^,^48^,^49^. The band at 1638 cm^−1^ was attributed to the bending vibration (δ_H_2_O_) of the H_2_O molecules in the interlayers^1^,^45^. A weak band at 1360 cm^−1^ corresponds to mode ν_3_ of the interlayer carbonate species^1^, which was a contaminant from the ambient atmosphere. More importantly, the absorption band at 1095 cm^−1^, which was attributed to the asymmetric vibration of PO_4_ 50,51,52, was clearly identified in the spectra of the HTln samples after the phosphate uptake experiments, indicating that the phosphorus was successfully intercalated into the interlayers of HTln, accompanied by the release of Cl^−^ (as previously shown in Fig. 3). Mg-Fe-**Cl** HTln also adsorbed fat, as revealed by the bands at 2923, 2850 and 1746, which are characteristic of fat and associated with vibrations of C–H and C=O bonds^53^,^54^. These bands were observed in the spectrum of the dried milk, revealing that the fat was from the milk.

**Figure 8:**
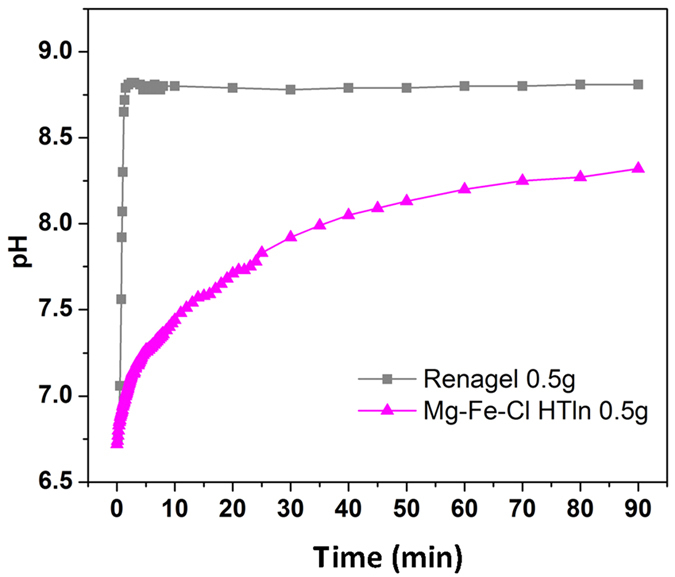
pH variation of 0.5 g Mg–Fe–Cl HTln and 0.5 g Renagel in 25 ml milk as a function of time.

Figure 8 plots the evolution of the pH of 25 ml milk (the milk pH was not maintained) during in the uptake of phosphorus by 0.5 g Renagel and 0.5 g Mg-Fe-Cl HTln, respectively. The pH curve of the milk with the Renagel rose dramatically from 6.7 to 8.8 in 3 min, and was subsequently flat until the end of the experiment. However, the pH curve of the milk with the Mg-Fe-**Cl** HTln gently increased over time, taking 90 min to reach pH 8.2. This result is attributable to the pH-buffering effect of the HTln^39^,^55^,^56^. The increase in the alkalinity of the solution with Renagel was much more aggressive than that with Mg-Fe-**Cl** HTln. Hur *et al*.^57^ showed that any increase in pH during gastric digestion may limit peptic degradation. Renagel has been reported to have gastrointestinal side effects (nausea, vomiting, abdominal pain, bloating, diarrhea, and constipation) in 38% of patients^11^,^20^, perhaps because of the rapid increase in pH and its extensive swelling (Fig. 4) when taken with liquid. Under the pH conditions shown in Fig. 8, according to ICP-AES analysis, 0.5 g Mg-Fe-Cl HTln could uptake ~11% of the phosphorus from the milk in 30 min and ~22% of it at 90 min. Although Renagel removed 30% of the phosphorus from milk in 30 min, but restoration of the phosphorus in the milk was observed beyond 30 min, finally ~26% phosphorus uptake was found at 90 min.

### Evaluation of *in-vitro* cytotoxicity of Mg-Fe-Cl HTln

**Figure 9:**
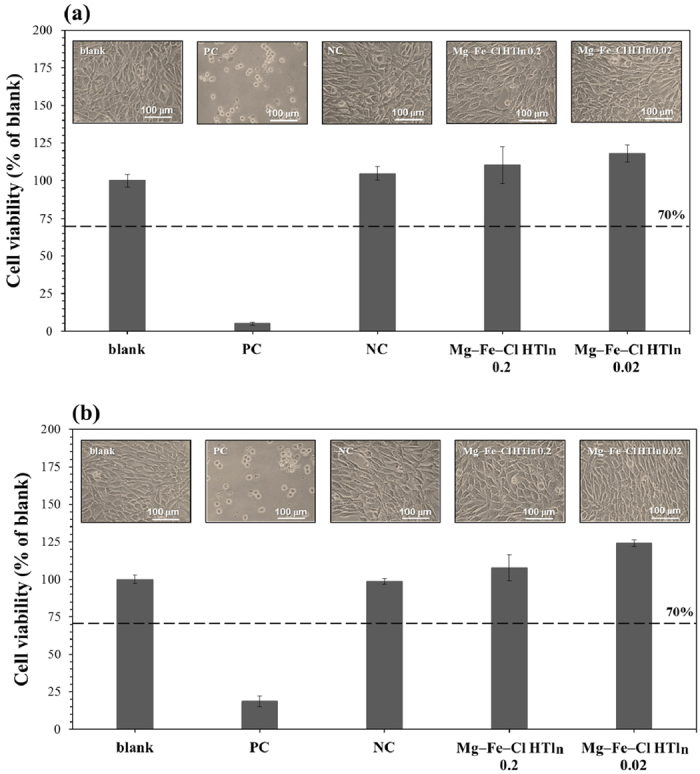
The morphology and viability of L919 cells after culturing for 24 h in Dulbecco’s modified Eagle’s medium (DMEM) that was obtained from 0.02 g/ml and 0.2 g/ml of extracts of Mg-Fe-Cl HTln powder immersed in the DMEM for 10 min (**a**) and 12 h (**b**). As shown, blank is blank control, PC is positive control, and NC is negative control.

Figure 9 presents the morphology and viability of L919 cells after culturing for 24 h in DMEM that contained different doses 0.02 and 0.2 g/ml of extracts of Mg-Fe-**Cl** HTln powder: Fig. 9(a,b) refer to extracts that were obtained following the immersion of Mg-Fe-**Cl** HTln powder in the medium for 10 min and 12 h, respectively. As displayed in Fig. 9(a,b), regardless of the extract concentration, L929 cells that were cultured in the extract-containing media were not morphologically different from, and were even slightly more viable than, the cells that were cultured in extract-free media (blank control). These results reveal that the Mg-Fe-**Cl** HTln powder extracts in this study were potentially non-cytotoxic. In clinical applications, phosphate binders in tablet form (0.8–1.6 g) are taken three times per day with food. Under the assumption that the tablets are taken with 50 ml water, then the concentration of the phosphate binder is 0.016–0.032 g/ml, which is close to the Mg-Fe-**Cl** HTln powder extract dose 0.02 g/ml that was used herein. A higher Mg-Fe-**Cl** HTln powder extract dose of 0.2 g/ml was also used to simulate an excessive dose. In this investigation, the phosphate binder was assumed to remain in the human body for less than 6 h (the interval between meals). Mg-Fe-**Cl** HTln powder extract was obtained after immersion for 12 h, which is the period between dinner and breakfast the following day, whereas Mg-Fe-**Cl** HTln powder extract was obtained after immersion for 10 min, which corresponds to the rapid absorption of the phosphate binder during a meal. As presented in Fig. 9, no evidence of cytotoxicity of the Mg-Fe-**Cl** HTln powder was obtained, even when the dose of Mg-Fe-**Cl** HTln powder or exposure duration exceeded that anticipated for clinical applications. Notably, cell viability in the experimental groups exceeded that in the blank control group. Reducing the concentration of Mg-Fe-**Cl** HTln powder extract from 0.2 g/ml to 0.02 g/ml would increase cell viability.

The human body requires approximately 250–500 mg Mg daily to maintain physiological processes and the healthy function of cells; the average 70 kg human body contains about 20 g of Mg^58^. Mg ions have been shown to have a marked effect on the phenotype of osteogenic cells both *in vivo* and *in vitro*^59^,^60^. Fe ions are also essential for metabolic processes, including oxygen transport^61^. Research has established that pure Fe extracts have negligible cytotoxic effects on human endothelial cells^62^. As presented in Fig. 9(b), the fact that Mg-Fe-**Cl** HTln powder improved cell viability (*vs*. blank group) is partially attributable to the positive effects of metallic ions (mainly Mg ions) on cell response. Reducing the Mg-Fe-**Cl** HTln powder extract dose from 0.2 g/ml to 0.02 g/ml further increased cell viability. According to the ICP-MS analysis, the concentrations of Mg and Fe ions in the Mg-Fe-**Cl** HTln powder extract (dose 0.2 g/ml; immersion time 12 h) were approximately 1800 ppm and 8 ppm, respectively. The Mg-Fe-**Cl** HTln powder extract at a dose 0.02 g/ml and immersion time of 12 h yielded an Mg ion concentration of around 200 ppm and no detectable Fe ions. This result reveals that approximately <1800 ppm Mg ions have a positive effect on cell viability, whereas a lower concentration (approximately 200 ppm) of Mg ions has an even more positive effect. Nonetheless, the mechanism that underlies the effects of Mg ions that are released from Mg-Fe-**Cl** HTln powder on cell viability requires further investigation.

## Methods

### Synthesis and characterization of Mg-Fe-Cl HTln powder

Mg(OH)_2_ and FeCl_3_·6H_2_O powders were utilized to synthesize Mg-Fe-Cl HTln. In Table 1, A represents Mg(OH)_2_ and B represents FeCl_3_·6H_2_O. Three powder samples, each containing A and B, denoted as 2A_0.1B, 2A_0.4B and 2A_0.6B. Each powder sample was immersed in 1000 ml distilled water at 50 °C. The pH of the aqueous solution was adjusted to, and maintained at, pH 1 by adding HCl_(aq)_ to totally dissolve the chemicals. The pH of the ionic solution was then increased up to 9.5 by adding NaOH_(aq)_ (2.5 M) dropwise. When the pH value was stable at pH 9.5 at 50 °C, a reddish-brown suspension was present in the solution. The solution pH was maintained at pH 9.5 for 2 hr at 50 °C. The above mixture was vigorously stirred magnetically and bubbled by forcing 1 LPM Ar gas into the aqueous solution throughout the synthesis. The solution with the reddish-brown suspension was then extracted using a centrifuge, rinsed five times with distilled water, and then vacuum dried.

The crystallographic structures of synthetic products were identified by X-ray powder diffraction using a Bruker D2 Phaser diffractometer. Ni filtered Cu *K*α1 (1.5406 Å) radiation was used for this purpose. The synthetic products also underwent Fourier transform infrared (FT-IR) analysis on a Perkin-Elmer Spectrum RX-I spectrometer. For the FT-IR analysis, 0.002 g HTln powder, mixed with 0.2 g oven-dried (80 °C, 1 h) spectroscopic-grade KBr, was pressed into a disc of diameter 12.91 mm under 8 tons of pressure for one minute in a vacuum. FT-IR spectra at wavenumbers of 400 to 4000 cm^−1^ were obtained. Elemental chemical analyses of synthetic products were performed by X-ray Photoelectron Spectroscopy (XPS). A JEOL JSM-7000F field emission scanning electron microscope was used to observe the topography of the synthetic products.

### Anionic sorption and desorption of Mg-Fe-Cl HTln in aqueous KH_2_PO_4_

Aqueous KH_2_PO_4_ (100 ml), comprising about 1000 ppm PO_4_^3−^, was used to evaluate the ability of the synthesized Mg-Fe-Cl HTln powder to take up PO_4_^3−^ from the KH_2_PO_4_ aqueous. In each experimental run, 0.2 g of the Mg-Fe-Cl HTln powder was immersed in 100 ml aqueous KH_2_PO_4_. Each solution in the experiment was purged with argon to reduce the formation of carbonate anions from the atmospheric gaseous CO_2_. The concentrations of Cl^−^ and residual PO_4_^3−^ in the aqueous KH_2_PO_4_ were simultaneously measured using ion chromatography (IC; ICS-900, DIONEX). Dilute nitric acid (2 vol.%) was used to maintain the pH of the aqueous at pH 3.0 ± 0.2 or pH 6.0 ± 0.2 throughout the anion sorption and desorption experiment. The error in the PO_4_^3−^ concentration that arose from the addition of aqueous nitric acid to maintain the solution pH was less than 5%.

### Mg-Fe-Cl HTln powder and sevelamer hydrochloride (Renagel) in cow milk and characterization thereof following phosphorus uptake experiments

The phosphate binding performance of Mg-Fe-Cl HTln powder was compared with that of a commercial phosphate binder, sevelamer hydrochloride (Renagel) in cow milk. To 25 ml cow milk were added 0.5 g Mg-Fe-Cl HTln or 1 g Mg-Fe-Cl HTln or 1 g of sevelamer hydrochloride powder. The original pH of the milk was around 6.7. Two experimental approaches were used to study the effect of pH on the ability of the HTln to take up phosphorus. In the first, the pH of the milk was adjusted to pH 6.0 ± 0.2 by adding aqueous HNO_3_ (50 vol.%), and this pH value was consistently maintained throughout the phosphorus uptake experiment. In the second, the milk pH was not maintained at a constant value during the phosphorus uptake experiment. Concentrations of residual phosphorus (mg/kg, ppm by mass) in the milk were measured by inductively coupled plasma atomic emission spectroscopy (ICP-AES) using US EPA method 3050B. The above quantitative analyses were carried out by Chemical Laboratory-Taipei, SGS TAIWAN LTD.

X-ray powder diffraction was used to compare the Mg-Fe-Cl HTln before phosphorus uptake with that after phosphorus uptake. Fourier transform infrared (FT-IR) analyses were performed to obtain the spectra of both Mg-Fe-Cl HTln and sevelamer hydrochloride (Renagel) before and after the uptake of phosphorus in cow milk.

To measure the volume changes of Mg-Fe-Cl HTln and sevelamer hydrochloride (Renagel) after each of the binders was mixed with cow milk for 90 min, a 25 ml capacity graduated cylinder with graduation marks every 0.5 ml was used. A 0.5 g mass of Mg-Fe-Cl HTln or 0.5 g sevelamer hydrochloride (Renagel) was mixed with 7 ml milk in the cylinder. The initial volume was read right immediately after mixing. The volume was read again after 90 min. The volume change (Δ V%) was thus obtained.

### *In-vitro* cytotoxicity assay of Mg-Fe-Cl HTln powder

L929 cells from a mouse fibroblast cell line were used to study the cytotoxicity of Mg-Fe-Cl HTln powder, according to ISO 10993-5 specifications. Mg-Fe-Cl HTln powder was immersed in Dulbecco’s modified Eagle’s medium (DMEM) (0.02 and 0.2 g/ml) in an incubator under 5% CO_2_ at 37 °C for different durations (10 min and 12 h). The concentrations (in parts per million (ppm)) of Mg and Fe ions in extracts were analyzed by inductively coupled plasma-mass spectrometry (ICP-MS) with a detection limit of 0.001 ppm for Mg ions and 0.044 ppm for Fe ions. Extracts were then used to treat a cell monolayer for 24 h, and then the cells were examined for morphological changes to assign toxicity scores. Cell viability was evaluated using 3-(4,5-dimethylthiazol-2-yl)-2,5-diphenyl tetrazolium bromide (MTT) assay and the optical density (OD) was measured using a microplate photometer (wavelength 570 nm): higher OD values indicated greater cell viability. The base medium (DMEM) without extract was used as a blank control; DMEM that had been treated with 10% dimethyl sulfoxide was used as a positive control (PC), and a biomedical grade zirconia sheet was used as a negative control (NC). If the cell viability was less than 70% of that of the blank group (medium only), then the extract was considered to be potentially cytotoxic.

## Additional Information

**How to cite this article**: Lung, Y.-F. *et al*. Synthesis of Mg-Fe-Cl hydrotalcite-like nanoplatelets as an oral phosphate binder: evaluations of phosphorus intercalation activity and cellular cytotoxicity. *Sci. Rep*. **6**, 32458; doi: 10.1038/srep32458 (2016).

## Figures and Tables

**Table 1:** Mg(OH)_2_ and FeCl_3_·6H_2_O for preparing Mg–Fe–Cl HTln. The compounds that would be produced hereafter were also denoted.

A	B	Designation of the compound produced hereafter
Mg(OH)_2_	FeCl_3_·6H_2_O	
2 g	0.1 g	2A_0.1B
2 g	0.4 g	2A_0.4B
2 g	0.6 g	2A_0.6B

**Table 2:** Mg^2+^, Fe^3+^, Cl^−^ and Na^+^ contents in the Mg–Fe–Cl HTln as evaluated by XPS.

	Composition/at.%				atomic ratio from the compound	
	Mg^2+^	Fe^3+^	Cl^−^	Na^+^	(Mg^2+^: Fe^3+^)	x
2A_0.1B	16.9	3.1	0.8	ND.	5.5 : 1	0.16
2A_0.4B	14.6	4.0	1.2	ND.	4.1 : 1	0.22
2A_0.6B	13.4	4.8	2.3	ND.	2.8 : 1	0.26

*where x = (Fe^3+^)/(Mg^2+^+Fe^3+^).
